# The Influence of CNC Turning with VBMT, RCMX, 3ER, and MGMN Type Indexable Inserts on West African Ebony/*Diospyros crassiflora*, San Domingo Boxwood/*Phyllostylon brasiliense*, Rio Rosewood/*Dalbergia nigra*, Beechwood/*Fagus sylvatica*, Oakwood/*Quercus robur*, and Pinewood/*Pinus silvestris* Surface Roughness

**DOI:** 10.3390/ma14195625

**Published:** 2021-09-27

**Authors:** Michał Bembenek, Rafał Kudelski, Jan Pawlik, Łukasz Kowalski

**Affiliations:** Faculty of Mechanical Engineering and Robotics, AGH University of Science and Technology, A. Mickiewicza 30, 30-059 Kraków, Poland; kudelski@agh.edu.pl (R.K.); jan.pawlik@agh.edu.pl (J.P.); lkowalski@agh.edu.pl (Ł.K.)

**Keywords:** TC inserts, surface roughness quality, CNC woodturning, West African Ebony/*Diospyros crassiflora*, San Domingo Boxwood/*Phyllostylon brasiliense*, Rio Rosewood/*Dalbergia nigra*, Pinewood/*Pinus silvestris*, Beechwood/*Fagus sylvatica*, Oakwood/*Quercus robur* surface roughness

## Abstract

The current scientific literature lacks an adequate description of hardware used to machine timber. Traditional woodworking and metals fabrication consists of tungsten carbide (TC) inserts. In this work, the authors investigate the influence of cutting tool geometry on the resulting surface roughness of timber samples. A variety of wood types were used in these studies to provide broad information on the correlation between the cutting tools used and resulting surface morphology. The cutting tools were prepared on a computer numerical control (CNC) lathe and roughness average (R_a_) and average maximum peak to valley height of the profile (R_z_) parameters were measured by contact stylus. The tip radius of the TC inserts used was determined to be the most significant factor that impacted R_a_ and R_z_. In summary, we found that the tip radius of the TC insert was inversely proportional to the roughness level, indicating that a flatter TC insert cutting end results in a smoother wood surface.

## 1. Introduction

Since Neolithic times, toolmakers have sought the best possible geometry for tools used in shaping lumber into the desired design [1,2]. A giant leap has since taken place in woodworking, as CNC machines are broadly replacing hand guidance and SiO_2_ flints are being replaced by precisely manufactured tungsten carbide (TC) cutting tools with a variety of different geometries [3,4,5,6]. TC inserts were primarily designed to work with metal, and there currently exist no CNC cutting tools manufactured specifically for woodworking. Some manufacturers tend to use steel or HSS steel tools, but, according to Zelinka et al. and Winkelmann et al., it carries a risk of tool corrosion, owing to, inter alia, tannins inside the structure of the particular lumber species [7,8].

The method of obtaining a complex shape and good repeatability of the fabricated elements is oftentimes achieved through application of CNC mills [9], CNC routers, numerically controlled water abrasive cutters [10], CNC lathes, or special veneer peeling lathes [11]. One of the parameters used to assess the quality of machining is the surface roughness [12,13,14,15]. Roughness not only plays the key role in positive feedback from the human user/end-user [16,17], but also provides information about the level of dimensional accuracy in the metrological aspect, especially in business sectors that require narrow tolerances—for instance, manufacturing of musical instruments [18,19]. Naturally, surface roughness of an intermediate timber product may be improved by adding an extra post-processing step to the production pipeline (for instance—sanding). The drawback of this approach is a visible impact on the general manufacturing process duration, costs, and possible influence on dimensional accuracy [20,21]. The sanding process is also not welcome in the veneer manufacturing process, as it strongly affects the veneer thickness [22]. In 2015, Owusu et al. conducted an experiment showing that some of the hardwood species, like *Diospyros mespiliformis* (similar to the ebony wood studied by our team), required slightly more time and effort than the other wood species in order to achieve a smooth surface finish, which makes sanding an even more expensive step [23].

In 2013, Hiziroglu et al. studied the role of the magnitude of roughness parameters in the bonding strength of boards of several different wood species, and it appeared that, in the glued joints in woodworking, the rough profile plays an additional role of interlocking the bonded surfaces by slightly raising the bonding strength [24]. That being stated, it appears that minimal surface roughness is not always the ultimate goal.

Regarding the surface roughness of a machined wood, it happens that approaches and methods of measurement are somewhat discrepant. Approaches taken while assessing wooden products’ surface roughness and quality vary between researchers.

Some researchers, like Morala-Argüello et al., use vision systems and wavelet transform, which belongs to the category of optical means of roughness measurement [25], while others, like Zhong et al. or Thoma et al., conducted the measurements with contact stylus methods [13,21,26]. In 2017, Gurau et al. performed an analysis of the pros and cons of those two approaches in their review [27] and recommended contact means of measurement where possible, even though they noticed that, hitherto, there are no official standards for performing the measurement of the roughness of the wooden surface. That is partially explained by Thibaut et al. in their interesting article from 2015, containing a summary of 50 years of wood machining science (focused on, but not limited to France) [28]. Thibaut discovered that, every year, the scientists produce several times more research papers about metalworking than about woodworking.

The craftsmen of the woodturning domain manage their choice of xylotheque with a high level of proficiency, yet the modern literature about woodturning is very scarce, as the studies on roughness usually refer to metal and metal alloy parts. The aforementioned Thoma et al. studied the roughness of the particular wood species after processing them on a planning machine by measuring the chip thickness [26]. In 2014, Sofouglu used the Taguchi method and ANOVA approach while determining the optimal cutting parameters of wooden edge glued pine panels, milled on a three-axis CNC router [29]. The same method was also implemented by Hazir, Koc et al. in 2019 in another effort of analyzing the influence of cutting parameters on the surface roughness in CNC routing, and they proposed another good model for estimation [30]. The most recent studies of roughness of machined wood were performed in 2021 by Ayanleye et al.—using an approach based on spectroscopy and modelling a fuzzy logic neural network, enabling prediction of the final roughness based on the lumber structure [31]; however, they did not take woodturning into account.

Other attempts to study the influence of machining parameters on the surface roughness were performed by Rawangwong et at. concerning exotic coconut wood [32] and palm wood [33]. Sofuoglu et al. in 2015 measured the impact of machining parameters on another four different species: european black pine, black poplar, sessile oak, and lebanonese cedar [34]. The conclusion from their studies was as follows: while keeping the cutting parameters the same, for every type of wood, the expected surface roughness parameters may vary. In this paper, the authors aim to address the lack of published studies on the influence of the type of the cutting tool geometry and the cutting depth on the surface roughness quality of six types of lumber (heretofore unexamined) in woodturning. Three of the species are European and the other three are considered as exotic wood. One of the goals of this paper is to estimate the possible impact of TC cutting tool insert geometry on the expected surface roughness of six different wood species without adding an extra post-processing step. Our hypothesis for this paper is as follows: there is a minimal level of the expected surface roughness, which is connected with the tip radius of the cutting tool.

## 2. Materials and Methods

**Figure 1:**
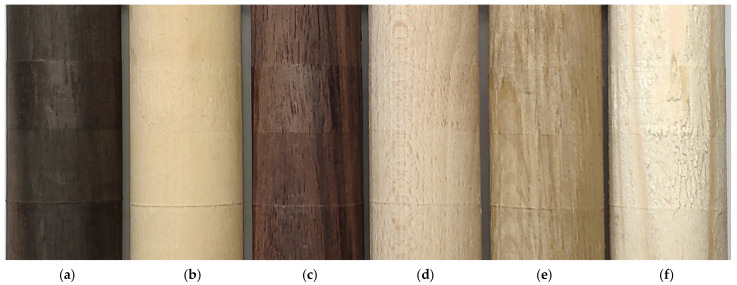
The wood specimens used in the tests (**a**) West African Ebony, *Diospyros crassiflora* (EB); (**b**) San Domingo Boxwood, *Phyllostylon brasiliense* (BoW); (**c**) Rio Rosewood, *Dalbergia nigra* (RW) (**d**) Beechwood, *Fagus sylvatica* (BeW); (**e**) Oakwood, *Quercus robur* (OW); (**f**) Pinewood, *Pinus silvestris* (PW).

To carry out the experiment, three exotic as well as three European specimens were prepared as thick wooden dowels with 35 mm of cross-section diameter and length of 280 mm. The specimens of exotic wood were selected in such a way that they have a high level of hardness (Janka index over 8000 N). Those wood types are used in manufacturing, i.e., violins and other musical instruments, and their color differs strongly. European wood species were selected based on their different density and hardness and, additionally, all of them are widely used in the timber-related industry including furniture or construction. Specimens were made out of the following species (Figure 1):

- West African Ebony, *Diospyros crassiflora* (EB);
- San Domingo Boxwood, *Phyllostylon brasiliense* (BoW);
- Rio Rosewood, Dalbergia nigra (RW);
- Beechwood, *Fagus sylvatica* (BeW);
- Oakwood, *Quercus robur* (OW);
- Pinewood, Pinus silvestris (PW).

**Figure 2:**
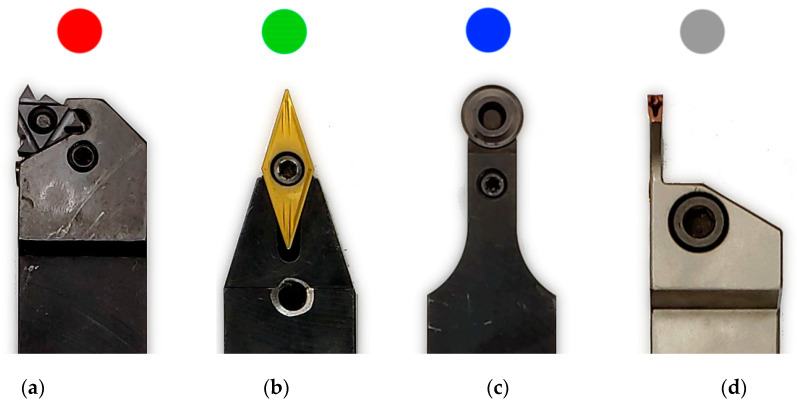
The indexable inserts used in the experimentL (**a**) 3ER threadcutting insert (TH), (**b**)VBMT pointed straight insert (PT), (**c**) RCMX round insert (RD), and (**d**) MGMN grooving insert (GR).

Afterwards, each one of the mentioned specimens was processed by turning on five-axis Takisawa-Nex 908 CNC (Takisawa, Taoyuan City, Taiwan) turning center, using four selected types of tungsten-carbide indexable inserts (Figure 2):

- 3ER threadcutting insert (TH);
- VBMT pointed straight insert (PT);
- RCMX round insert (RD);
- MGMN grooving insert (GR).

In order to better present the test results, the knives were assigned appropriate colors red, green, blue, and grey. The most important geometrical parameters of cutting inserts used needed to reproduce the experiment results are shown in Table 1. The indexable inserts were selected so that their tips have a different radius. For processing, specimens were mounted inside milling machine with three jaw chuck on the spindle side, and with a revolving centre on the other end.

**Figure 3:**
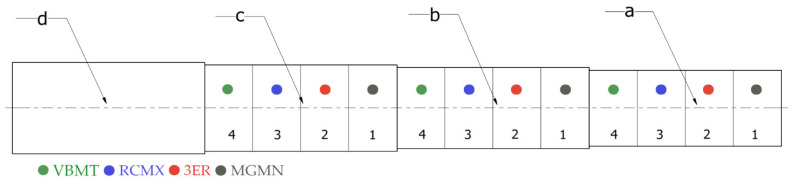
The specimen machining depth and tools zone (**a**) with 1.5 mm cutting depth; (**b**) with 1.0 mm cutting depth; (**c**) with 0.5 mm cutting depth; and (**d**) the unprocessed part reserved for clamping. Grooving insert (**1**), threadcutting insert (**2**), round insert (**3**), and pointed straight insert (**4**).

After being mounted, each specimen was virtually divided into three equally long zones alongside the axis of rotation, for three different depths of cut—0.5 mm, 1.0 mm, and 1.5 mm. Each zone was divided into four subzones, with the first being machined with before mentioned grooving insert, the second with threadcutting insert, the third with round insert, and fourth with pointed straight insert. Before cutting with the designated depths of cut, each zone was first cut with 1.0 mm depth of cut using a zone-specific tool, in order to eliminate possibility of lathe chuck runout influencing the results and reproduce the real turning process as accurately as possible. Figure 3 shows schematically how the turning process was carried out. For the entire process, the same cutting speed of 200 m/min and feedrate of 0.2 mm/rotation were maintained. The same process turning was repeated for each type of wood examined in this study. A total of 72 turning tests were performed.

**Figure 4:**
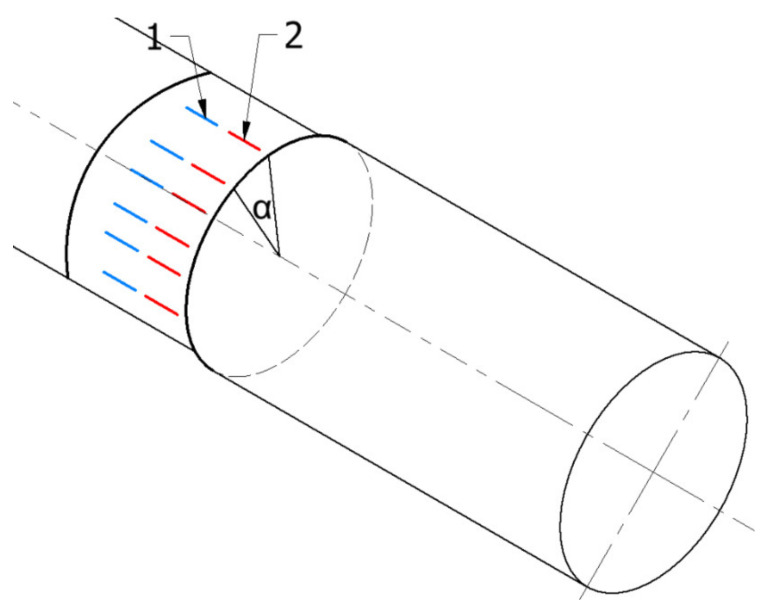
Schematically shown roughness measurement a series for one subzone (one tool type and cutting depth) (**1**), (**2**)—place of measurements, (**α**)—rotating angle.

After preparing the specimens, surface roughness of the processed areas was measured accordingly, using surface roughness measuring system Mitutoyo Formtracer SV-C3000 (Mitutoyo, Kanagawa, Japan) with measurement head SV-C4500 (Mitutoyo, Kanagawa, Japan). Arithmetic average roughness (R_a_) and mean roughness depth (R_z_) as per ISO 4287 were measured on each subzone at 12 different measurement lines, parallel to the wood grain direction and perpendicular to the cutting direction. For clarification, Figure 4 shows the measurement methodology for one subzone.

**Figure 5:**
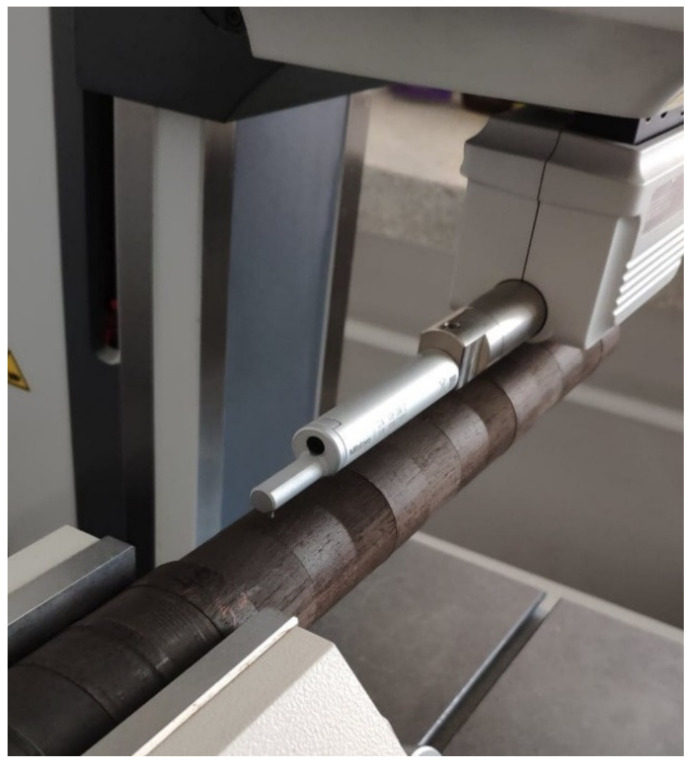
Placement of measurement head’s needle on examined *Diospyros crassiflora* specimen.

Two measurements were carried out parallel to wood grain direction, then the specimen was rotated by 30 degrees for another pair of measurements, repeating the process until obtaining 12 roughness profiles for each subzone, totaling 144 measurements for each wood type, which gave 864 measurements in total. To obtain accurate results, while measuring round stocks roughness, the measured track was tangent to the specimen cross section (Figure 5).

Each roughness profile was 4.8 mm long, sampled with a 0.0005 mm step, and measured at a stylus movement speed of 0.5 mm/s. The measurement settings were dictated by the default settings of the measuring system, as the evaluation length matched the size of the samples. The multiple regression analysis was performed using the Analysis ToolPak in Microsoft Office 2010. The R_a_ roughness results were used to build a model describing the relationship between the Janka hardness (JH), the depth of cutting (CD), and insert corner radius (ICR). For each type of the wood, the following hardness values were assessed for the analysis [35]:

- West African Ebony 13,700 N;
- San Domingo Boxwood 8050 N;
- Rio Rosewood 12,410 N;
- Beechwood 6460 N;
- Oakwood 4980 N;
- Pinewood 2420 N.

In order to perform the analysis, it was initially assumed that the radius of the MGMN insert would be 100 times the feed and would be 20 mm. In reality, the tip radius of MGMN tends to infinity, being a flat-end; however, the assumed value (20 mm) is nevertheless much higher (3–250 times) than the tip radii of other TC inserts, used in this experiment.

The wooden specimens were seasoned over a one year duration in enclosed area with air average humidity of 45% ± 30% and constant average temperature of 20 °C ± 3 °C. The moisture of the wood was determined from chips of each material by the weight method at 105 °C until a constant weight was obtained. The Vibra AJH 420 CE (Tokyo, Japan) scale was used. For each wood species, two measurements were carried out.

Macroscopic measurements were made with a Keyence VHX-7000 microscope (Keyence, Osaka, Japan) on the surface after turning. The captured area was 2.3 × 1.7 mm.

## 3. Results

The obtained results of the moisture content of wood specimens are as follows:

- West African Ebony 4.2%;
- San Domingo Boxwood 6.0%;
- Rio Rosewood 4.9%;
- Beechwood 3.5%;
- Oakwood 6.1%;
- Pinewood 6.2%.

They show that, in all sample cases, their moisture content was low and they were accurately dried to be mechanically processed.

In all cases of turning, the cylinder surfaces were properly drawn, hence it can be concluded that, in all cases, the parameters of the turning process were selected correctly. The obtained surface after turning was free from cracks or tears. The inserts used for turning were not damaged and showed no signs of visible wear. The worst visual surface quality was obtained for pine, which can be associated with the low hardness of this type of wood.

**Figure 6:**
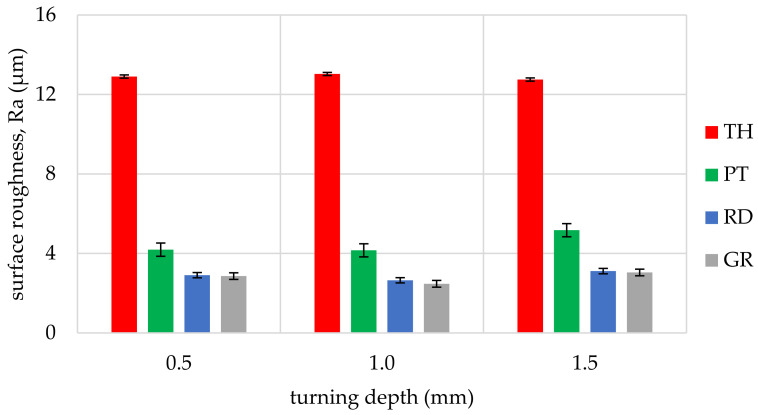
Influence of turning depth and type of cutting insert on surface roughness of West African Ebony, Diospyros crassiflora (EB).

**Figure 7:**
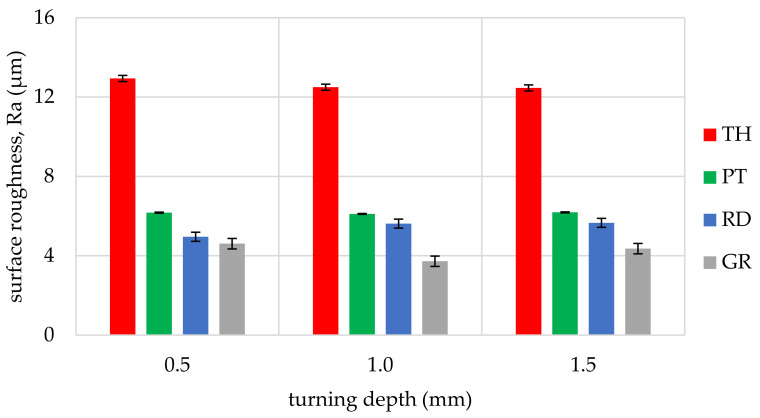
Influence of turning depth and type of cutting insert on surface roughness of San Domingo Boxwood, Phyllostylon brasiliense (BoW).

**Figure 8:**
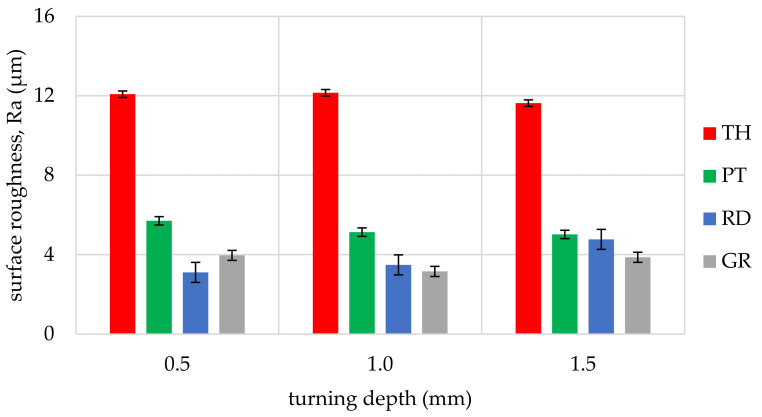
Influence of turning depth and type of cutting insert on surface roughness of Rio Rosewood, Dalbergia nigra (RW).

**Figure 9:**
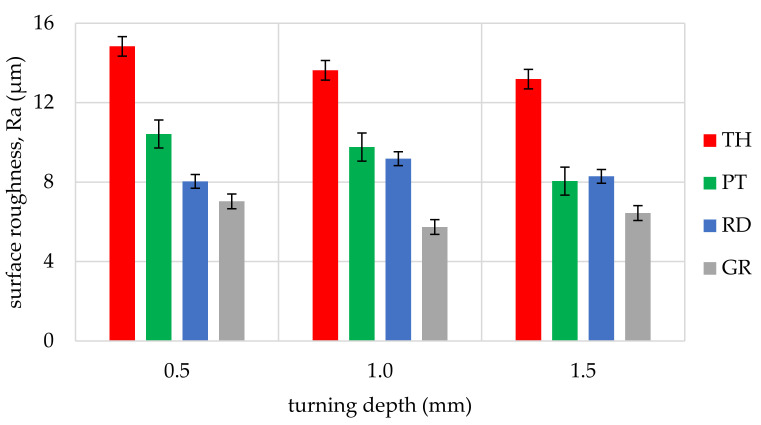
Influence of turning depth and type of cutting insert on surface roughness of Beechwood, Fagus sylvatica (BeW).

**Figure 10:**
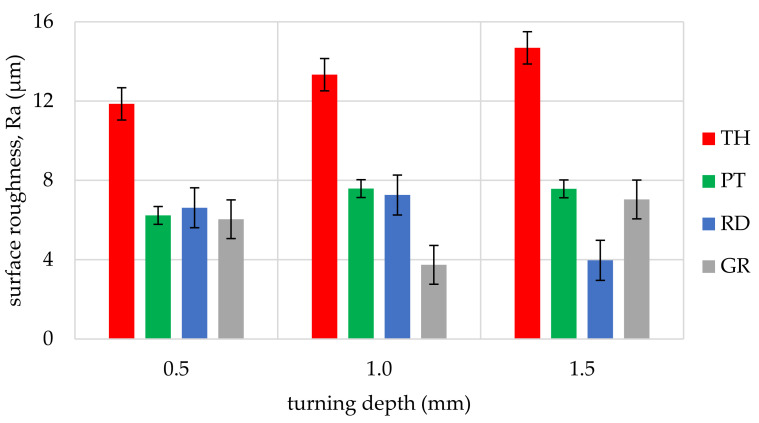
Influence of turning depth and type of cutting insert on surface roughness of Oakwood, Quercus robur (OW).

**Figure 11:**
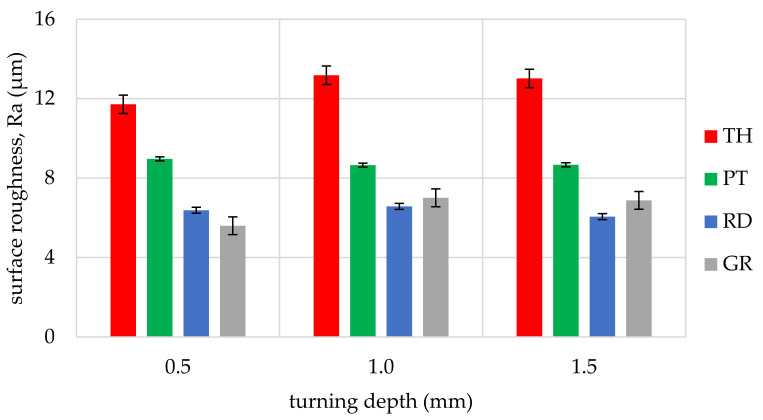
Influence of turning depth and type of cutting insert on surface roughness of Pinewood, Pinus silvestris (PW).

**Figure 12:**
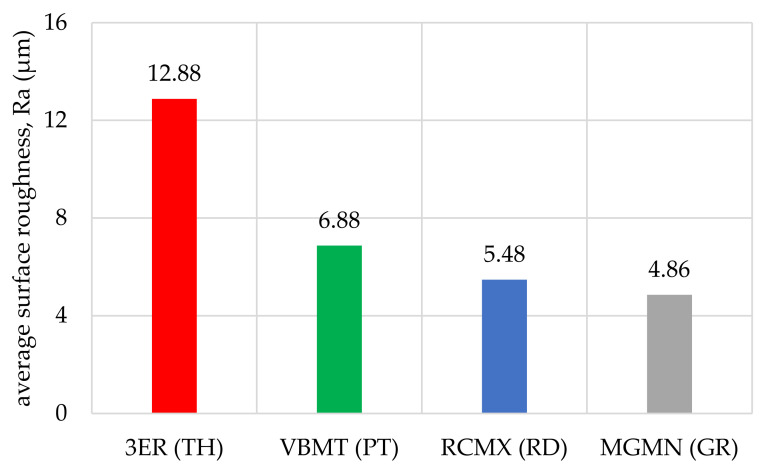
Average surface roughness R_a_ obtained in all tests depending on the insert used.

The obtained values of arithmetic average roughness (R_a_) are shown in Figure 6, Figure 7, Figure 8, Figure 9, Figure 10 and Figure 11 and in Table 2. In Figure 12, the average surface roughness R_a_ obtained in all tests depending on the insert used is shown.

**Figure 13:**
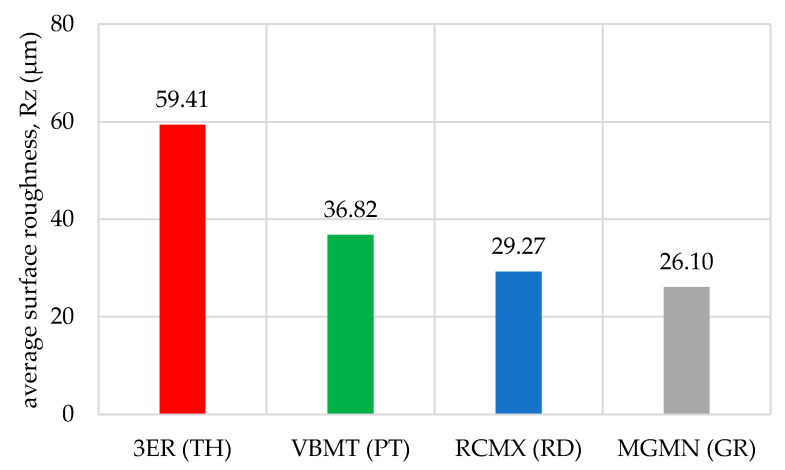
Mean roughness depth R_z_ obtained in all tests depending on the insert used.

**Figure 14:**
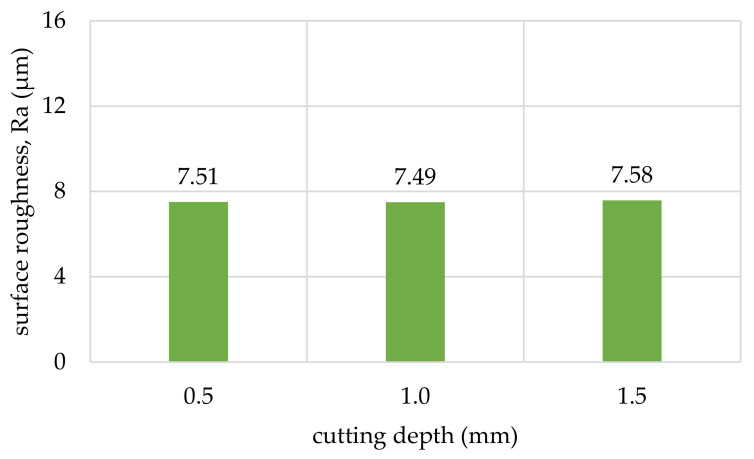
Average surface roughness obtained in all tests depending on the cutting depth.

**Figure 15:**
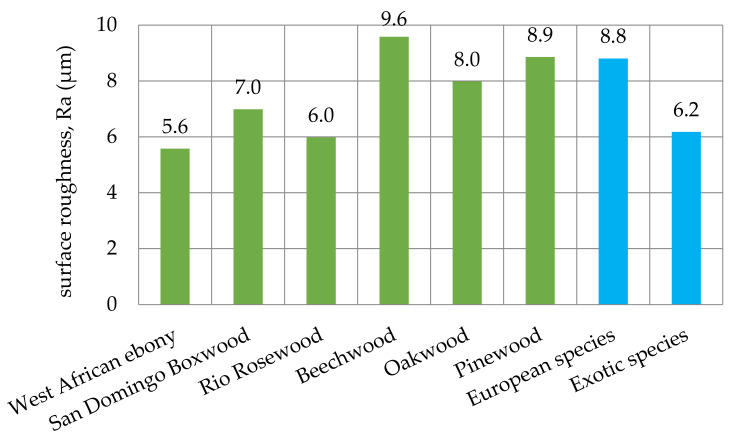
Average surface roughness obtained in all tests depending on the wood species.

**Figure 16:**
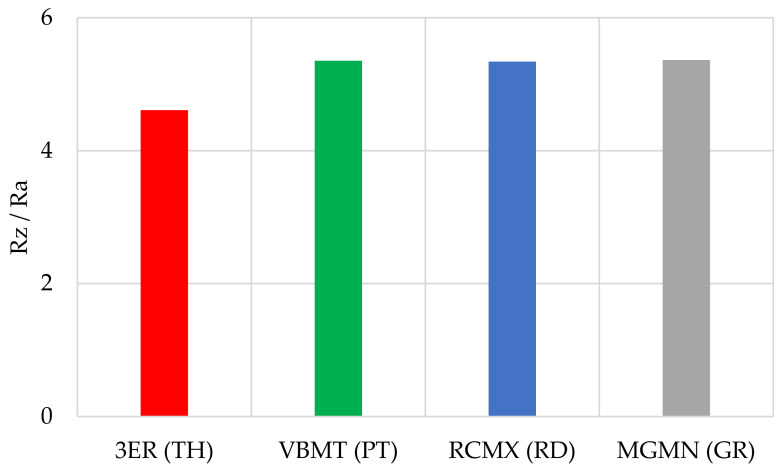
The quotients R_z_ to R_a_ obtained in all tests depending on the insert used.

**Figure 17:**
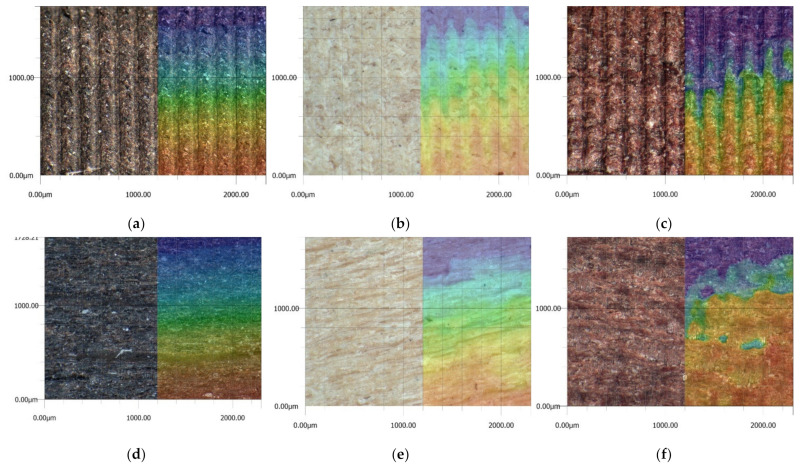

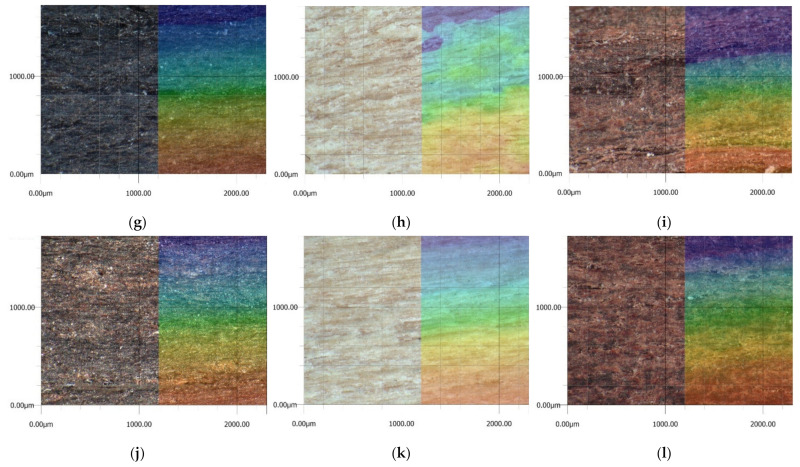
The macroscopic view of the surface after turning of a depth of 1.0 mm: (**a**) 3ER (TH) West African Ebony, (**b**) 3ER (TH) San Domingo Boxwood, (**c**) 3ER (TH) Rio Rosewood, (**d**) VBMT (PT) West African Ebony, (**e**) VBMT (PT) San Domingo Boxwood, (**f**) VBMT (PT) Rio Rosewood, (**g**) RCMX (RD) West African Ebony, (**h**) RCMX (RD) San Domingo Boxwood, (**i**) RCMX (RD) Rio Rosewood, (**j**) MGMN (GR) West African Ebony, (**k**) MGMN (GR) San Domingo Boxwood, and (**l**) MGMN (GR) Rio Rosewood.

**Figure 18:**
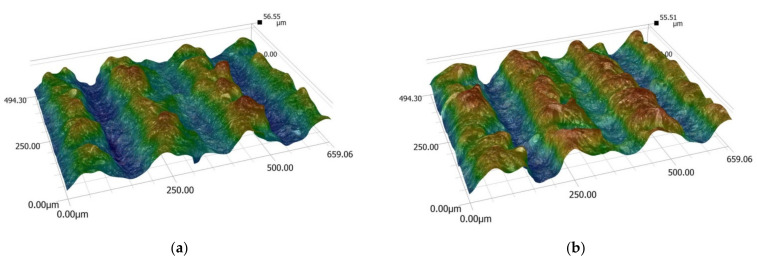

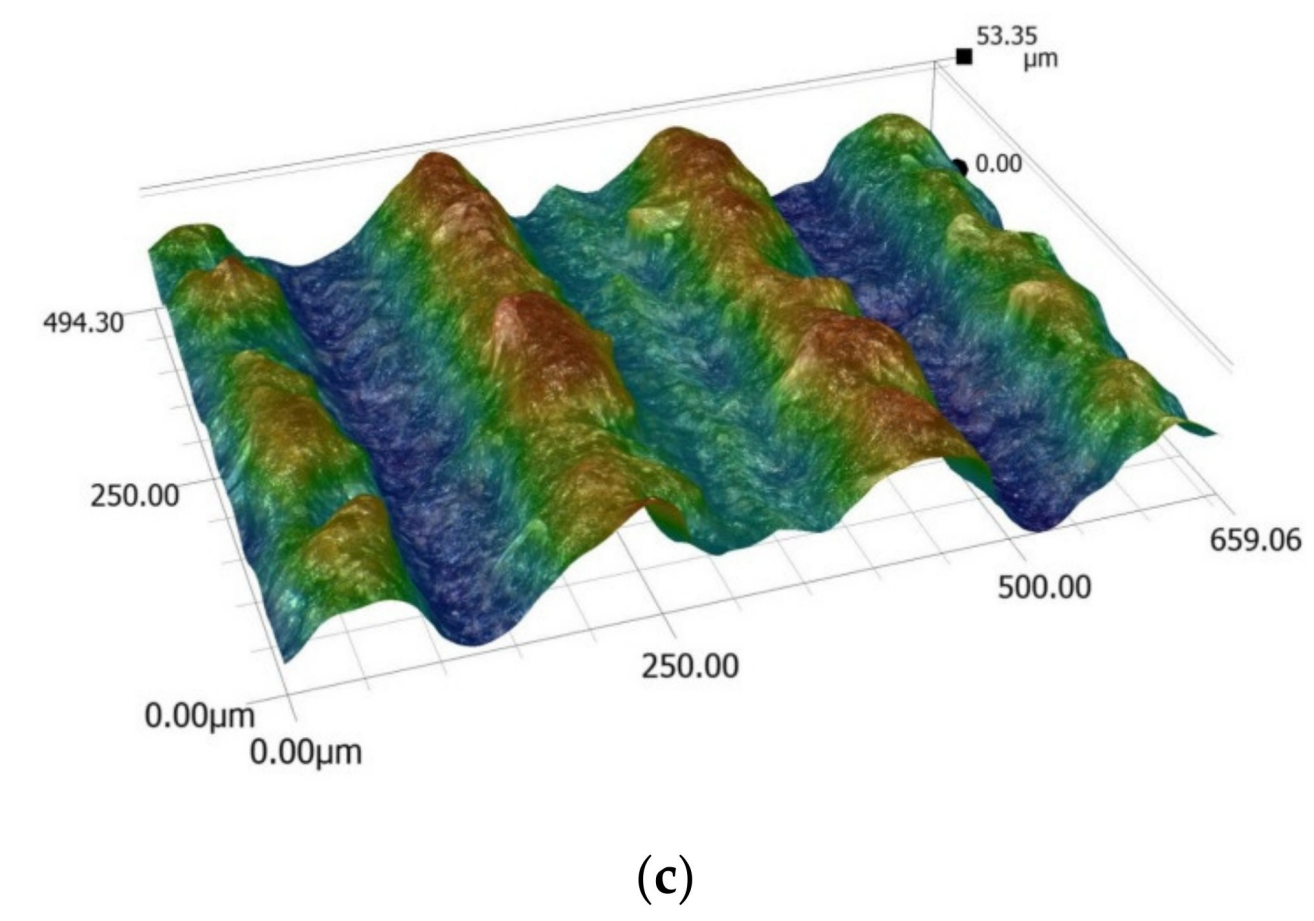
The macroscopic view of the surface of West African Ebony after turning with 3ER (TH) with a depth of (**a**) 0.5 mm, (**b**) 1.0 mm, and (**c**) 1.5 mm.

**Figure 19:**
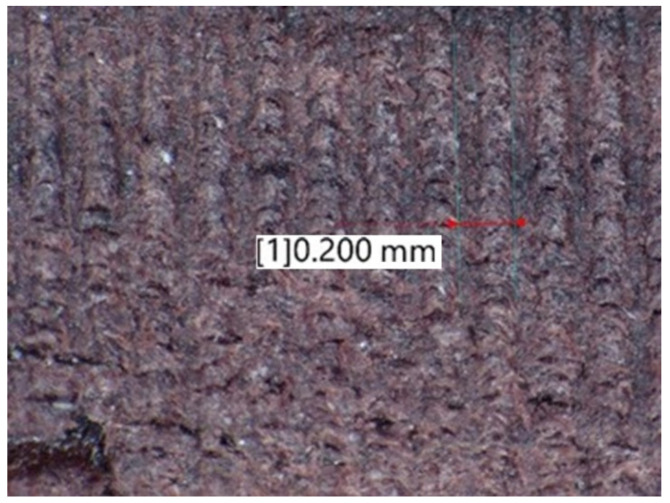
The macroscopic view of the surface of West African Ebony after turning with 3ER (TH) with an ap = 1.0 mm with the marked distance between successive depressions on the structure.

The obtained values of mean roughness depth R_z_ are shown in Figure 13 and in Table 3. Average surface roughness obtained in all tests depending on the cutting depth is shown in Figure 14. It was also determined that the quotient Rz to Ra depends on the type of insert and wood species. Their values are shown in Table 4 as well as in Figure 15. The sample macroscopic views of the surface after turning of a depth of 1.0 mm are shown in Figure 16. The macroscopic views of the surface of Pinewood after turning with 3ER (TH) with different depths are shown in Figure 17. The macroscopic views of the surface of West African Ebony after turning with 3ER (TH) with the differed depths as well as with the marked distance between successive depressions on the structure are shown in Figure 18 and Figure 19.

Initial multiple linear regression analysis showed the *p*-value for the cutting depth parameter was 0.929. Therefore, it can be concluded that the cutting depth parameter does not have a significant impact on the R_a_ value. On this basis, another analysis was carried out, excluding the cutting depth parameter. Its results are presented in Table 5. The conducted analysis showed that the assumed radius of the MGMN insert at the level of 20 mm was selected correctly, as the models built for the radius of a greater value showed less compliance with the test results.

## 4. Discussion

The contact methods utilized in the experiment turned out to be the proper way to measure the surface roughness of the machined wooden samples. The authors confirm the conclusions made by Gurau et al. in 2017 [27], as optical methods of measurement of such specific material (compound fibres, some half-transparent, some with high luster) are prone to be heavily distorted by incorrect settings or even ambient light. The experimental results obtained proved the correlation between the surface roughness of machined wood type and the geometry of the tool used.

The most repeatable results in full range of cutting depths (0.5–1.5 mm) were obtained for West African Ebony (Figure 6), indicating its quality in terms of processability and ability to achieve good surface finish without postprocessing. The R_a_ indicator value for ebony ranged from 2.47 µm to 5.17 µm (excluding the 3ER tool insert). The highest roughness values in the full range of cutting depths and inserts used were measured for Beechwood (Figure 9), which may originate from the wood structure itself [36]. The machined pole made out of BeW achieved R_a_ = 10.42 µm (excluding 3ER insert, for which Ra = {13,19; 13.63; 14,84 µm}), and that value might be out of the subjective scope of the aforementioned haptic comfort; therefore, this type of wood may require additional post-processing.

By analysing and comparing the data contained in Table 1, Table 2 and Table 3, it can be concluded that, while selecting the proper tool for the woodturning process, one has to take into account the following phenomenon—the expected level of roughness is inversely proportional to the tip radius of the cutting tool insert. Therefore, the smaller the radius, the higher the resultant R_a_, R_z_, and other parameters are obtained, and vice versa—as the dimension value for the tip of the tool increases—comparing, for example, the TH insert with a radius of 0.08 mm and clearance angle of 7° to the GR insert with a virtually infinite tip radius (Table 1) and no clearance—the surface of the machined element is smoother. This phenomenon is visible in the experiments results (Figure 6, Figure 7, Figure 8, Figure 9, Figure 10, Figure 11, Figure 12 and Figure 13), where the lowest R_a_ and R_z_ values were obtained while turning with a round (R_amin_ = 2.65 µm, R_zmin_ = 15.47 µm) or grooving insert (R_amin_ = 2.47 µm, R_zmin_ = 14.29 µm), and the highest values of surface roughness were obtained for a TH insert (R_amax_ = 14.84 µm, R_zmax_ = 70.09 µm). By comparison between the tool types used (Figure 12), the TH insert (the highest mean R_a_ value measured) is 62.27% larger than the R_a_ determined for the GR insert (the lowest R_a_ average measured). To compare, the mean values for second parameter calculated in this study, R_z_ (Figure 13), differ by a smaller percentage between TH and GR inserts, specifically by 56.07%. Differences between roughnesses of areas turned with other examined tools were less visible. Between second highest (PT) and lowest (GR) measured mean roughnesses, R_a_ differs by 29.36% and R_z_ by 29.11%. Between RD and GR inserts, R_a_ differs by 11.31% and R_z_ by 10.83%. From the practical perspective, said values allow to obtain good turning results with both GR and RD inserts, slightly in favor of the GR insert. The aforementioned percentage differences between R_a_ and R_z_ are fairly similar, which leads to conclusion that both coefficients describe the processed wood surface in a comparable manner. Statistical average roughness for the scope of tools used in tests on different wood types discussed shows superiority of GR and RD inserts over PT, and especially TH tools, in terms of the quality of surface finish after turning without postprocessing, as the average of measured roughness for TH is 2 to 2.5 times higher than average for the GR insert (Figure 12 and Figure 13). However, the TH insert has other advantages, namely it might be useful to the manufacturers that produce non-cylindrical wooden parts—for instance, spheres, spheroids, or other decorative motifs. In certain cases, it would be difficult or impossible to machine a sphere with, for example, a grooving insert.

Such results can be explained by the relation between the insert geometry and tool federate speed during the process, as the contact between the groove-cutting insert and particular areas on the surface of the timber was maintained for several workpiece rotations. In our experiment, the feedrate was set to 0.2 mm per rotation, and the tip radius of the thread-cutting insert was also 0.2 mm, thus it was possible to create a quasi-micro-thread, impacting surface roughness (Figure 19). This is visible on the charts above—when the 3ER insert was used, the R_a_ parameter was kept on almost the same level, despite the difference in the wooden material type processed.

The average roughness (from all tool types used) of European wood species is 30% lower than exotic wood species examined in this study (Figure 15). The best mean roughness was calculated for EB (5.6 µm), while the highest roughness was found for BeW (9.6 µm). Such a phenomenon has to be researched further, but might be correlated to the climate of the zone in which examined trees grow—areas with more stable weather conditions might allow the tree to grow more uniform with compacted interwoven layers of lignin and cellulose fibers.

The experimental data, followed by our statistical analysis, has shown that, concerning the surface roughness, the cutting depth (a_p_) is not as relevant as the selection of the proper insert geometry (Figure 14 and Figure 18), excluding the exceptions, being fibrous oakwood. The differences in the average roughness levels between a_p1_ = 0.5 mm, a_p2_ = 1 mm, and a_p3_ = 1.5 mm of the same types of wood were insignificant (seen on Figure 14), with the standard error at 0.027%, calculated from the whole dataset, for all a_p_. It is also visible in Figure 18, in microscopic view, that thread-like waves created by the TH insert do not differ in relation to cutting depth. As the structure of the wooden material is non-homogenous, the profile of the surface roughness tends to be influenced by the topology of interwoven fibers of the heartwood and the sapwood. This is especially visible in the case of the *Dalbergia Nigra* (rosewood). It was noticed that, only for oakwood (Figure 10), where Ra measured for the RD insert and depth of cut equal to 1.5 mm was visibly smaller than for 0.5 and 1.0 mm depths measured for the same tool—it was 39–45% smoother, respectively.

The determined quotient R_z_ to R_a_ ranges from 4.27 to 5.84. The lowest average quotient was obtained for the 3ER insert (4.61), and it differed from other inserts by about 14%. For the others, the quotient was very similar and amounted to approximately 5.35. The performed statistical analysis allowed for the development of a model of the process described using the following formula:

$$R_{a}=-0.00028\cdot JH-0.25046\cdot ICR+11.41135$$

where

R_a_—surface roughness, µm;
JH—Janka hardness, N;
ICR—insert corner radius, mm.

The best correlation of the measurement results with the estimated model is obtained for the PT, RD, and GR inserts.

## 5. Conclusions

The conducted multiple linear regression analysis showed that the cutting depth parameter does not have a significant impact on the R_a_ value.

The experiments also show the following practical conclusion: while machining simple, cylindrical shapes in order to achieve the lowest surface roughness parameters, the best choice is a groove-cutting insert. Unfortunately, it was noted that the market lacks commercially available angled cutting tool holders for the GR inserts—perhaps because of the fact that the GR insert is not designed specifically for woodturning with low roughness values. Yet, when the designed shape is not a cylinder (for instance a sphere or an ornamental thread), one might rather consider using the round shaped insert; nevertheless, the size of the tip radius is in this case constrained by the complexity of the non-cylindrical geometry.

The conducted experiments have shown that the achievable level of surface roughness is inversely proportional to the hardness of the wood, hence the average roughness of European wood species used in tests is approximately 30% higher than exotic wood species examined in this study, keeping other parameters the same. In order to obtain high roughness of the wood surface, for example, to glue it (as stated Hiziroglu et al. [24], the high roughness of the surfaces to be glued affects the high strength of the joint), it is recommended to treat this type of surface before gluing with the TH insert. The presented test pieces were prepared on a CNC lathe, but the conclusions are also eligible for conventional, manual lathes.

## Figures and Tables

**Table 1:** Basic geometrical parameters of used cutting inserts.

Insert Type	Color	Symbol	Corner Radius, mm	Tool Orthogonal Wedge Angle, °	Clearance Angle, °
3ER threadcutting insert (TH)	red	3 ER AG60 VTX	0.08	60	7
VBMT pointed straight (PT)	green	VBMT160404-MF2CP500	0.4	35	5
RCMX round insert (RD)	blue	RCMX 12 04 00 4225	6.0	15	-
MGMN grooving insert (GR)	grey	N123G2-0300-0002-CM-1125	0.2 ^1^	-	-

^1^ Right and left edge.

**Table 2:** The R_a_ values obtained in the tests (with standard error value in the brackets) depend on the type of insert and wood species.

Species of Wood	Turning Depth, mm	Type of Insert/Surface Roughness R_a_, µm			
		3ER (TH)	VBMT (PT)	RCMX (RD)	MGMN (GR)
West African Ebony *Diospyros crassiflora* (EB)	0.5	12.90 (±0.33%)	4.19 (±0.25%)	2.91 (±0.33%)	2.86 (±0.41%)
	1.0	13.03 (±0.39%)	4.16 (±0.24%)	2.65 (±0.17%)	2.47 (±0.28%)
	1.5	12.75 (±0.27%)	5.17 (±0.39%)	3.11 (±0.33%)	3.04 (±0.22%)
San Domingo Boxwood *Phyllostylon brasiliense* (BoW)	0.5	12.94 (±0.44%)	6.17 (±0.37%)	4.96 (±0.38%)	4.61 (±0.26%)
	1.0	12.50 (±0.31%)	6.11 (±0.23%)	5.62 (±0.27%)	3.72 (±0.27%)
	1.5	12.46 (±0.34%)	6.19 (±0.39%)	5.66 (±0.39%)	4.36 (±0.22%)
Rio Rosewood *Dalbergia nigra* (RW)	0.5	12.08 (±0.32%)	5.70 (±0.38%)	3.11 (±0.42%)	3.96 (±0.51%)
	1.0	12.15 (±0.40%)	5.13 (±0.36%)	3.48 (±0.50%)	3.16 (±0.47%)
	1.5	11.63 (±0.28%)	5.02 (±0.37%)	4.77 (±0.59%)	3.86 (±0.86%)
Beechwood *Fagus sylvatica* (BeW)	0.5	14.84 (±0.52%)	10.42 (±0.92%)	8.04 (±0.86%)	7.03 (±0.64%)
	1.0	13.63 (±0.43%)	9.77 (±0.99%)	9.18 (±1.06%)	5.74 (±0.66%)
	1.5	13.19 (±0.43%)	8.05 (±0.53%)	8.29 (±0.93%)	6.44 (±0.62%)
Oakwood *Quercus robur* (OW)	0.5	11.86 (±0.84%)	6.23 (±0.60%)	6.62 (±1.38%)	6.04 (±1.38%)
	1.0	13.34 (±0.86%)	7.59 (±0.91%)	7.26 (±1.19%)	3.74 (±0.55%)
	1.5	14.68 (±0.89%)	7.57 (±0.92%)	3.97 (±0.43%)	7.04 (±1.20%)
Pinewood *Pinus silvestris* (PW)	0.5	11.72 (±0.47%)	8.97 (±0.96%)	6.38 (±0.57%)	5.60 (±0.66%)
	1.0	13.18 (±0.68%)	8.66 (±1.06%)	6.58 (±0.81%)	7.01 (±0.82%)
	1.5	13.02 (±0.73%)	8.67 (±0.84%)	6.06 (±0.85%)	6.88 (±0.49%)
All average	0.5–1.5	12.88	6.88	5.48	4.86

**Table 3:** The R_z_ values obtained in the tests depend on the type of insert and wood species.

Species of Wood	Turning Depth, mm	Type of Insert/Surface Roughness R_z_, µm			
		3ER (TH)	VBMT (PT)	RCMX (RD)	MGMN (GR)
West African Ebony *Diospyros crassiflora* (EB)	0.5	56.32 (±1.67%)	23.54 (±1.41%)	16.62 (±1.72%)	16.38 (±2.26%)
	1.0	55.68 (±1.24%)	23.80 (±1.22%)	15.47 (±0.92%)	14.29 (±1.43%)
	1.5	54.74 (±1.07%)	29.37 (±2.43%)	17.03 (±1.58%)	17.47 (±1.25%)
San Domingo Boxwood *Phyllostylon brasiliense* (BoW)	0.5	56.18 (±2.04%)	33.52 (±1.63%)	27.01 (±2.18%)	25.90 (±1.51%)
	1.0	57.13 (±1.71%)	32.64 (±1.21%)	29.58 (±1.59%)	20.79 (±1.27%)
	1.5	55.52 (±1.94%)	33.28 (±1.79%)	31.77 (±2.34%)	24.10 (±0.99%)
Rio Rosewood *Dalbergia nigra* (RW)	0.5	54.99 (±1.55%)	30.56 (±1.81%)	17.64 (±2.39%)	21.05 (±2.31%)
	1.0	56.62 (±1.75%)	28.91 (±1.79%)	19.67 (±2.51%)	18.40 (±2.56%)
	1.5	51.91 (±1.26%)	28.85 (±2.13%)	26.05 (±3.22%)	21.07 (±3.49%)
Beechwood *Fagus sylvatica* (BeW)	0.5	69.09 (±2.00%)	54.48 (±5.39%)	42.56 (±3.86%)	37.53 (±2.88%)
	1.0	65.08 (±2.60%)	52.79 (±4.62%)	47.12 (±4.22%)	31.10 (±3.34%)
	1.5	62.75 (±2.57%)	44.88 (±2.85%)	41.39 (±4.11%)	32.26 (±2.80%)
Oakwood *Quercus robur* (OW)	0.5	55.15 (±3.61%)	34.00 (±2.81%)	34.46 (±5.89%)	33.13 (±7.03%)
	1.0	63.42 (±4.05%)	39.15 (±3.66%)	39.47 (±6.18%)	20.88 (±2.76%)
	1.5	70.09 (±4.72%)	40.14 (±4.22%)	21.92 (±2.22%)	37.30 (±5.16%)
Pinewood *Pinus silvestris* (PW)	0.5	56.53 (±1.93%)	45.90 (±4.86%)	32.84 (±3.02%)	27.98 (±2.99%)
	1.0	62.78 (±4.12%)	43.95 (±4.59%)	34.54 (±4.08%)	35.57 (±3.55%)
	1.5	65.39 (±4.33%)	43.03 (±3.87%)	31.77 (±4.32%)	34.53 (±2.30%)
All average	0.5–1.5	59.41	36.82	29.27	26.10

**Table 4:** The quotients R_z_ to R_a_ obtained in the tests depend on the type of insert and wood species.

Species of Wood	Turning Depth, mm	Type of Insert/R_z_ to R_a_			
		3ER (TH)	VBMT (PT)	RCMX (RD)	MGMN (GR)
West African Ebony *Diospyros crassiflora* (EB)	0.5	4.37	5.62	5.71	5.73
	1.0	4.27	5.72	5.84	5.79
	1.5	4.29	5.68	5.48	5.75
San Domingo Boxwood *Phyllostylon brasiliense* (BoW)	0.5	4.34	5.43	5.45	5.62
	1.0	4.57	5.34	5.26	5.59
	1.5	4.46	5.38	5.61	5.53
Rio Rosewood *Dalbergia nigra* (RW)	0.5	4.55	5.36	5.67	5.32
	1.0	4.66	5.64	5.65	5.82
	1.5	4.46	5.75	5.46	5.46
Beechwood *Fagus sylvatica* (BeW)	0.5	4.66	5.23	5.29	5.34
	1.0	4.77	5.40	5.13	5.42
	1.5	4.76	5.58	4.99	5.01
Oakwood *Quercus robur* (OW)	0.5	4.65	5.46	5.21	5.49
	1.0	4.75	5.16	5.44	5.58
	1.5	4.77	5.30	5.52	5.30
Pinewood *Pinus silvestris* (PW)	0.5	4.82	5.12	5.15	5.00
	1.0	4.76	5.08	5.25	5.07
	1.5	5.02	4.96	5.24	5.02
All average	0.5–1.5	4.61	5.35	5.34	5.37

**Table 5:** The obtained results of the Janka hardness (JH) and insert corner radius (ICR) multiple linear regression analysis.

	Coefficients Value	Standard Error	t-Statistic	*p*-Value	Upper 95%	Lower 95%
Constant	11.41135	0.783334	14.56766	9.74 × 10^−23^	9.848638	12.97406
insert corner radius (ICR)	−0.25046	0.040501	−6.18413	3.86 × 10^−8^	−0.33126	−0.16967
Janka hardness (JH)	−0.00028	8.24 × 10^−5^	−3.3778	0.001204	−0.00044	−0.00011

## Data Availability

The data presented in this study are available on request from the corresponding author.
